# Orofacial lesions associated with long-term highly active antiretroviral therapy among HIV-seropositive adults in Ibadan, Nigeria

**DOI:** 10.11604/pamj.2021.38.370.25322

**Published:** 2021-04-15

**Authors:** Oluwatoyin Elizabeth Abe, Omotayo Francis Fagbule, Oluwatobi Olubusi Olaniyi, Akinyele Olumuyiwa Adisa, Olalere Omoyosola Gbolahan

**Affiliations:** 1 Department of Oral Pathology and Oral Medicine, University College Hospital, Ibadan, Nigeria,; 2 Department of Periodontology and Community Dentistry, University College Hospital, Ibadan, Nigeria,; 3 Infectious Disease Institute, College of Medicine, University of Ibadan, Ibadan, Nigeria,; 4 Department of Oral Pathology, Faculty of Dentistry, College of Medicine, University of Ibadan, Ibadan, Nigeria,; 5 Department of Oral and Maxillofacial Surgery, College of Medicine, University of Ibadan/ University College Hospital, Ibadan, Nigeria

**Keywords:** Highly active antiretroviral therapy, HIV/AIDS, HIV-seropositivity, orofacial lesions, oral candidiasis, oral ulcers, CD4 count, Nigeria

## Abstract

**Introduction:**

highly active antiretroviral therapy (HAART) has contributed to a reduction in HIV- related oral lesions and improved quality of life among HIV seropositive patients. However, the therapy is not without its side effects. This study was aimed at assessing the self- reported orofacial manifestations due to long term use of HAART, as well as the pattern of oral lesions on examination.

**Methods:**

this was a cross-sectional study conducted among HIV seropositive adult patients in Ibadan, who had been on HAART for at least two years. Data were collected using an interviewer-administered questionnaire. Clinical diagnosis of HIV-related oral lesions was made according to the EC-Clearinghouse criteria. Data analysis was done using SPSS version 25.

**Results:**

the study participants comprised of 227 HIV seropositive patients who were HAART experienced, with 54 (24%) males and 173 (76%) females. Their mean age (±SD) was 44.7 (±9.4) years. The participants CD4 count ranged from 13-1338cells/mm^3^, with a median count of 341 cells/mm^3^. About half (45%) of the participants noted one or more orofacial changes since they commenced HAART. These oral changes included dryness of mouth, burning sensation, abnormal taste, melanotic hyperpigmentation, oral thrush, ulcers, and parotid swelling. Most of those who reported oral changes had been on HAART over 10 years (p=0.03), and the changes were more reported among those on the first-line regimen.

**Conclusion:**

melanotic hyperpigmentation was the most common oral lesion found and burning mouth syndrome was the most commonly reported complain among HIV-seropositive adults who are on long-term HAART.

## Introduction

Highly active antiretroviral therapy (HAART) has become a standard treatment for HIV infection globally and has been found to induce marked reduction in viral load and an increase in the CD4+ cell count [1]. The introduction of HAART into the management of HIV has been associated with improved clinical and laboratory outcomes, reduction in morbidity and mortality and overall improved quality of life among people living with HIV/AIDS (PLWHA) [2, 3]. Although HAART initiation has enhanced the treatment of HIV/AIDS infection, the therapy is associated with some systemic adverse effects including orofacial effects [3]. The orofacial adverse effects of HAART are oral ulcers, xerostomia, mucositis, hyperpigmentation, erythema multiforme (EM), toxic epidermal necrolysis, lichenoid reactions, cheilitis, perioral paresthesia, angioedema, and taste alteration [4].

Nucleotide reverse transcriptase inhibitors (NRTIs) such as Zidovudine can cause bone marrow suppression, which may predispose to oral ulcers, oral lichenoid reaction, and mucositis [5]. Non-nucleoside reverse transcriptase inhibitors (NNRTIs) especially Nevirapine, has been associated with severe cutaneous manifestations, such as Stevens-Johnson syndrome and toxic epidermal necrolysis [5, 6]. The protease inhibitor drugs can induce lipodystrophy syndrome; consisting of abnormal fat distribution, central adiposity, insulin resistance, hyperglycemia, and hyperlipidaemia [7, 8]. This lipodystrophy condition could present in the orofacial region as parotid lipomatosis and facial lipo-atrophy. Taste abnormalities have also been implicated in protease inhibitors such as indinavir, ritonavir, and saquinavir [8]. The medications usually have a bitter, metallic, astringent and sour taste, as well as burning sensation [8]. The effect of HIV infection is profound on the salivary glands, both on its anatomic structure and physiologic function [9, 10]. Some classes of HAART, especially the protease inhibitors (PIs), can cause salivary gland dysfunction; thus, leading to decreased salivary flow rates and features of xerostomia [9, 10].

The Federal Government of Nigeria introduced the national antiretroviral therapy (ART) program in 2002 with free drug provision to adults and children [5]. Currently, Nigeria is yet to meet the global target of enrolling 90% of people diagnosed with HIV infection to be on HAART; just 33% of them were on HAART as of 2017 [11]. Since the use of HAART is lifetime, varying regimen have been produced over the years to improve patients´ compliance with drug use and to minimize side effects [12]. With the ongoing use of HAART in Nigeria over the last eighteen years [5], there is a dearth of literature documenting possible effects of HAART on the orofacial tissues in Nigeria, compared to other parts of the world. Identifying possible side effects that may be associated with the different regimen will be a useful tool in the future production of an appropriate regimen for HIV seropositive patients in this environment. Therefore, this study was conducted to determine the pattern of orofacial lesions among HIV seropositive patients on HAART in Ibadan, Nigeria. Their commonly reported oral symptoms as related to HAART were also assessed.

## Methods

The study was a descriptive cross-sectional design conducted among HIV-seropositive patients attending the outpatient adult HIV clinic of the Infectious Diseases Institute, College of Medicine, University of Ibadan, Nigeria. Ethical approval was obtained from the University of Ibadan and University College Hospital, Ibadan Joint Ethical Review Committee. The study was conducted over a period of four months, from August to November 2018. The inclusion criteria were adults who had been on HAART for at least two years [10]. All consenting participants who met the inclusion criteria, who came for either collection of drugs or consultation were included in the study.

Data collection was done using an interviewer-administered questionnaire. Information obtained were socio-demographics, social habits (alcohol intake and smoking), co-morbidity, CD4 count, mode of HIV infection, duration, and type of antiretroviral therapy. Oral examination was carried out by two dental surgeons using facemasks, disposable wooden spatulas, and latex gloves under natural light. Clinical diagnosis of HIV-related oral lesions was made according to the criteria proposed by the EC-Clearinghouse [13]. The participants who had symptomatic oral lesions were referred to the tertiary dental clinic, which is in collaboration with the HIV clinic, for appropriate management.

Data analysis was done using SPSS version 25. Quantitative variables such as age and duration on HAART were summarized using means and standard deviation. Categorical variables such as gender, marital status, and oral lesion types were expressed as proportions. Pearson´s Chi-square test was used to assess the association between the independent variables and the outcome variables, and the level of significance was set at 5% (p<0.05).

## Results

The study participants comprised of 227 HIV-seropositive patients on HAART, consisting of 54 (24%) males and 173 (76%) females. Their ages ranged from 18- 80 years with a mean age of 44.7 (±9.4) years. The majority were married (63%), while more participants reported Christianity (64.3%) as their religion (Table 1). Only 15% of the participants reported co-morbid conditions like hypertension, diabetes mellitus, and peptic ulcer disease, which they were being managed for alongside their HIV status. Majority of the patients reported contracting the viral infection through heterosexual route (78%); others were through blood transfusion (2.2%), mother to child transmission (0.4%), men having sex with men (0.4%), and some were unknown (17.6%).

**Table 1:** socio-demographic characteristics of the study participants

Socio-demographic characteristics	Frequency (N=227)	Percentage (%)
**Gender**		
Male	54	23.8
Female	173	76.2
**Age group (years)**		
15-30	9	4.0
31-45	128	56.4
46-60	75	33.0
61-80	15	6.6
**Marital status**		
Single	16	7.0
Married	143	63.0
Separated/ Divorced	24	10.6
Widow	44	19.4
**Religion**		
Christian	146	64.3
Muslim	79	34.8
Missing	2	0.9
**Level of education**		
None	11	4.8
Primary	75	33.0
Secondary	75	33.0
Tertiary	66	29.2

**Figure 1:**
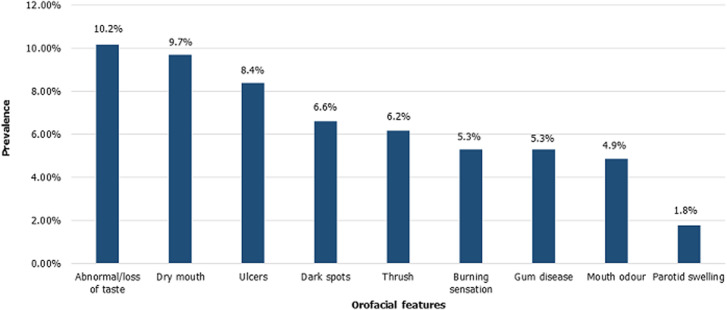
prevalence of self-reported orofacial problems experienced by HIV-seropositive adults following HAART use in Ibadan, Nigeria, 2018

Forty-five percent of the participants reported one or more oral changes since they commenced HAART. Some of the self-reported oral related changes were dryness of mouth, burning sensation, abnormal taste, dark spots, oral thrush, ulcers, and parotid swelling (Figure 1). More participants were on first-line drugs (67.6%) compared to the second-line drugs (32.4%) (Table 2). Most (77.2%) of those who reported oral changes had been on HAART for at least six years while a higher proportion of those on the first-line drugs (65%) reported more oral changes than those on second-line drugs (35%) (Table 2). Notably, features of burning mouth syndrome (dryness of mouth, burning sensation, and abnormal taste) were the most reported symptoms. Burning mouth syndrome was more frequently reported among females and by those on first-line HAART. Most of the participants who had been on HAART for over 10 years reported features of burning mouth syndrome compared to those on it for less than 10 years (p=0.03) (Table 3).

**Table 2:** factors associated with self-reported orofacial lesions among participants

Variable	Oral effect of HAART		p-value
	**Present (%)**	**Absent (%)**	
**Duration on HAART**			0.32
2-5 years	23 (22.8)	25 (20.0)	
6-10 years	40 (39.6)	62 (49.6)	
>10 years	38 (37.6)	38 (30.4)	
**HAART regimen**			0.46
First-line	65 (65.0)	85 (69.7)	
Second line	35 (35.0)	37 (30.3)	

**Table 3:** factors associated with the presence of burning mouth syndrome among the study participants

Variable	Burning mouth syndrome		p-value
	**Present (%)**	**Absent (%)**	
**Gender**			0.14
Male	15 (27.8)	39 (72.2)	
Female	32 (18.5)	141 (81.5)	
**HAART regimen**			0.39
First-line	28 (18.7)	122 (81.3)	
Second-line	17 (23.6)	55 (76.4)	
**HAART duration**			0.03*
2-5 years	13 (27.1)	35 (72.9)	
6-10 years	13 (12.7)	89 (87.3)	
>10 years	21 (27.6)	55 (72.4)	

* significant

Intra-oral examination revealed that 41% of the participants had at least one oral lesion; oral melanotic hyperpigmentation (19%) was the most common lesion. Others included candidiasis (13%), periodontal diseases (7%), oral ulcers (1%), and fibroma (1%). The mean (±SD) and median (inter-quartile range) CD4 count of the participants were 387.2 (±277.6) cells/mm^3^ and 341 cells/mm^3^ (IQR 13-1838) respectively. Almost half (48%) of the participants had CD4 count ranging from 200 cells/mm^3^ to 500 cells/mm^3^. The majority (77.8%) of respondent with CD4 count <500 cells/mm^3^ had oral lesions on examination (p=0.01) (Table 4).

**Table 4:** relationship between CD4 count and HAART regimen with the presence of oral lesions among the study participants

Variables	Oral lesions		p-value
	**Present (%)**	**Absent (%)**	
**CD4 count (cells/mm^3^)**			0.01*
<200	33 (36.7)	26 (19.0)	
200-500	37 (41.1)	72 (52.6)	
>500	20 (22.2)	39 (28.5)	
**HAART regimen**			0.09
First-line	55 (61.1)	95 (72.0)	
Second-line	35 (38.9)	37 (28.0)	

* significant

## Discussion

The oral findings in the present study align with those in earlier documented works that have assessed HIV-infected patients who were HAART experienced [9, 10, 14, 15]. Melanotic hyperpigmentation was the most common oral lesion seen among our study participants, which has been commonly documented among PLWHA from African and Asian countries [1, 16-19]. Other oral lesions like candidiasis, ulcers, and periodontal lesions found among our participants have also been reported [18, 20-23]. We found that two in every five of our study participants had at least one oral lesion. This suggests that orofacial symptoms are common problems among HAART-experienced people living with HIV/AIDS (PLWHA) in Nigeria. This burden may yet increase, considering that they have to be on HAART for life. It is therefore crucial that their oral health is prioritized alongside their medical care. The role of the oral healthcare professionals in improving and maintaining the oral health of PLWHA is pivotal for an acceptable quality of life; thus, the need for increased oral health promotion among PLWHA.

Also, about half of the study participants reported having witnessed some changes in their oral cavity since the time they commenced HAART. The most-reported oral symptom in long term use of HAART from this study was burning mouth syndrome (burning sensation, dysgeusia and xerostomia). This was found mostly among those who had been on HAART for more than 10 years. This finding is consistent with studies from India as reported by Nittayananta *et al*. [10] and Verma *et al*. [24] among HIV- infected respondents, on long term HAART. More of our participants on the first-line regimen of HAART reported having these oral symptoms, and this is consistent with the findings by Kumar *et al*. [9].

Despite the use of HAART for at least two years, among the respondents in this study, a high degree of immunosuppression was observed among them. Most of our respondents had CD4 count < 500 cell/mm^3^; almost half of these were severely immunosuppressed (CD4 <200 cells/mm^3^). This was significantly related to the presence of some HIV-related oral lesions, as documented. The level of immunosuppression found among our participants could be due to their inadequate adherence to HAART as well as poor nutrition due to low socioeconomic status. We suggest more extensive multi-center studies in Nigeria to investigate the relationship between CD4 count and orofacial lesions.

The study has its limitations; since the participants´ responses were self-reported, they are prone to misreporting bias. There is also the risk of recall bias, considering that some of them had been on HAART for several years. The study employed a cross-sectional design, therefore, causality and temporality could not be established; the identified orofacial lesions could be due to other causes apart from HAART.

## Conclusion

Burning mouth syndrome was the most recurring oral symptom associated with long-term use of HAART among HIV patients in Ibadan, Nigeria. As well, melanotic hyperpigmentation was the most commonly found orofacial lesion among them. A significant association was found between the presence of oral lesions and immunosuppression among the study participants.

### What is known about this topic

- Highly active antiretroviral therapy (HAART) helps to reduce HIV-associated lesions;
- HAART, like many other pharmacological agents, has its attendant side effects.

### What this study adds

- Identification of burning mouth syndrome as the most reported oral complaints among people living with HIV/AIDS (PLWHA) on long term HAART in Ibadan, Nigeria;
- Identification of melanotic hyperpigmentation as the most common orofacial lesion found among HAART-experienced PLWHA in Ibadan, Nigeria;
- Among PLWHA who are on long-term HAART in Ibadan, Nigeria, those with a CD4 count of <500 cells/mm^3^ are more likely than those with >500 cells/mm^3^ to have orofacial lesion.
